# Development and validation of the STeP score for predicting tracheostomy in patients with sepsis using a nationwide ICU database: a retrospective observational study

**DOI:** 10.1186/s40560-025-00833-8

**Published:** 2025-11-14

**Authors:** Kazuya Kikutani, Mitsuaki Nishikimi, Michihito Kyo, Satoshi Yamaga, Tatsutoshi Shimatani, Kohei Ota, Shinichiro Ohshimo, Nobuaki Shime

**Affiliations:** 1 Department of Emergency and Critical Care Medicine, Graduate School of Biomedical and Health Science, Hiroshima University, 1-2-3 Kasumi, Minami-ku, Hiroshima, 734-8551 Japan; 2 Department of Radiation Disaster Medicine, Research Institute for Radiation Biology and Medicine, Hiroshima University, Hiroshima, Japan

**Keywords:** Sepsis, Mechanical ventilation, Tracheostomy, Airway management

## Abstract

**Background:**

Among patients with sepsis admitted to the intensive care unit (ICU), a substantial proportion require mechanical ventilation, and a subset eventually undergo tracheostomy. Early identification of patients at high risk for tracheostomy may facilitate timely decision-making and improve clinical communication.

**Methods:**

We conducted a nationwide, retrospective study using the Japanese Intensive care PAtient Database (JIPAD). Adult patients with sepsis (Sequential Organ Failure Assessment score of ≥ 2, excluding viral pneumonia) who required mechanical ventilation between 2018 and 2022 were included. The primary outcome was tracheostomy within 14 days of ICU admission. Seventy-five variables available within 24 h of ICU admission were collected. Using least absolute shrinkage and selection operator (LASSO) regression with tenfold cross-validation, we selected predictors to build a multivariable logistic regression model (Sepsis Tracheostomy early Prediction [STeP] model). A simplified scoring system (STeP score) was also derived. Predictive performance was assessed using the area under the curve (AUC) of the receiver operating characteristic (ROC) in a temporally independent validation cohort.

**Results:**

Among 7357 eligible patients (training: 5374; validation: 1983), 1013 (13.8%) underwent tracheostomy. The STeP model, based on 8 LASSO-selected variables, demonstrated good discrimination (AUC: 0.76 in training, 0.74 in validation). The simplified STeP score (range, 0–17), derived from the same predictors, achieved an AUC of 0.73 in the validation cohort. Patients were stratified into low (≤ 2 points), moderate (3–6 points), and high (≥ 7 points) risk groups for tracheostomy, with corresponding tracheostomy rates of 4.0%, 13.6%, and 27.1%, respectively.

**Conclusions:**

We developed and validated a robust prediction model and simplified risk score (STeP score) for tracheostomy within 14 days in ICU patients with sepsis. Early risk stratification using variables available within 24 h may support timely tracheostomy planning. A web-based calculator is publicly available to facilitate bedside implementation.

**Supplementary Information:**

The online version contains supplementary material available at 10.1186/s40560-025-00833-8.

## Background

Sepsis is a critical condition encountered across all areas of healthcare—including emergency, intensive care, and general medicine—and is associated with high morbidity and mortality [1]. Its significance continues to grow with aging populations [2]. More than 40% of patients with sepsis require intubation and ventilator support for various reasons [3], including respiratory failure due to septic acute respiratory distress syndrome, septic shock, and impaired consciousness [4]. Some of these patients require a tracheostomy because of prolonged mechanical ventilation, decreased levels of consciousness, or difficulties with airway clearance related to impaired sputum expectoration. Tracheostomy plays a critical role not only in respiratory management but also in intensive care unit (ICU) resource utilization and patient outcomes. When appropriately timed, tracheostomy may facilitate earlier weaning from mechanical ventilation, reduce the ICU length of stay [5], promote earlier mobilization and rehabilitation, and enhance patient comfort by minimizing sedation requirements [6]. From a healthcare system perspective, timely tracheostomy can support more efficient resource allocation and cost reduction [7], especially in the context of increasingly strained critical care capacity.

Despite its importance, the decision to perform tracheostomy in patients with sepsis remains highly variable across clinicians and institutions. Given its significant impact on prognosis [8], ICU length of stay, and communication with families, early prediction of the need for tracheostomy is clinically relevant to a wide range of healthcare providers, including intensivists, emergency physicians, hospitalists, nurses, and caregivers. In addition to informing clinical decision-making, early prediction can also support ethical care by respecting patient autonomy and allowing families time to prepare for complex decisions.

Although numerous studies have investigated the prediction of tracheostomy in critical illness [9–15], only a limited number have specifically focused on patients with sepsis [16]. Moreover, in the context of sepsis, existing studies have primarily identified risk factors without developing a predictive model.

The aim of this study was to develop and validate a prediction model for tracheostomy within 14 days of ICU admission in patients with sepsis, using clinical information available within the first 24 h of ICU admission.

## Methods

### Study design and patient inclusion criteria

This retrospective observational study used data from the Japanese Intensive care PAtient Database (JIPAD), a nationwide database of critically ill patients admitted to ICUs in Japan. Detailed information about the JIPAD has been previously reported [17]. Briefly, the database prospectively collects data, including age, sex, comorbidities, blood pressure, body temperature, Glasgow Coma Scale scores, laboratory and blood gas data, use of mechanical ventilation and catecholamines, and severity scores, such as the Sequential Organ Failure Assessment (SOFA) and the Acute Physiology and Chronic Health Evaluation II (APACHE II). This study included patients who were urgently admitted to the ICU—not for planned surgery—between 1 April 2018 and 31 March 2023 (corresponding to Japanese fiscal years 2018–2022), were diagnosed with sepsis, and required mechanical ventilation. Sepsis was defined by a primary diagnosis code indicating infection [18] and a SOFA score of ≥ 2 [19]. Patients were excluded if they had already undergone tracheostomy at the time of ICU admission. Patients were also excluded if they had begun mechanical ventilation more than 24 h after ICU admission or had incomplete data—such as missing information on tracheostomy status at ICU admission or on mechanical ventilation. Patients whose primary diagnosis was viral pneumonia [18] were also excluded, because during the COVID-19 pandemic, there was a tendency to avoid or delay tracheostomy due to infection risk [20]. Patients who died in the ICU without undergoing tracheostomy were excluded as well, under the assumption that they may have required tracheostomy had they survived. This exclusion was intended to minimize bias and to focus the analysis on patients who had the potential to benefit from tracheostomy during their ICU stay. After applying these exclusion criteria, the patients were divided into a training cohort (2018–2021) and a validation cohort (2022). This study was approved by the Hiroshima University Institutional Review Board (IRB number: E2023-0274-02).

### Outcomes

The primary outcome was tracheostomy within 14 days of ICU admission.

### Model development and validation

In total, 75 variables were collected, including patient demographics, vital signs within the first 24 h of ICU admission, laboratory data, severity scores, and facility-level characteristics, such as the number of ICU-certified beds and dedicated nurses (Supplementary Table S1). Missing data among predictors ranged from 0.00% to 4.71% in the training cohort and from 0.00% to 4.03% in the validation cohort (Supplementary Table S2). Missing values were imputed using a non-parametric imputation method, applied separately to the training and validation data sets to maintain their independence.

To construct a parsimonious and interpretable model, we first applied logistic regression with L1 regularization, also known as least absolute shrinkage and selection operator (LASSO), using tenfold cross-validation to select candidate predictors from the 75 variables. Categorical variables were dummy-coded, with reference categories set as follows: normal body mass index (BMI) (for BMI groups), emergency department (for admission source), and no use (for vasopressor levels) (Supplementary Table S1). The optimal regularization parameter (lambda) was determined using the one-standard-error rule to yield more parsimonious models [21]. Subsequently, the variables selected by LASSO were used to fit a multivariable logistic regression model, which served as the final prediction model [22–24]. Model performance was evaluated in the independent validation cohort by calculating the area under the receiver operating characteristic curve (AUC). An AUC between 0.7 and 0.8 is considered to indicate good discrimination, while an AUC of > 0.8 is regarded as excellent [25]. Model calibration was additionally assessed using calibration plots and the Brier score. In the calibration plot, predicted probabilities were grouped into 10 fixed deciles, and the observed event rates within each bin were plotted. A calibration curve was fitted using locally estimated scatterplot smoothing with a 95% confidence interval (CI) to visualize the agreement between predicted and observed probabilities. To quantitatively assess calibration, the calibration slope and intercept were estimated by regressing the outcome on the logit-transformed predicted probabilities. Model development and validation were conducted in accordance with the Transparent Reporting of a multivariable prediction model for Individual Prognosis or Diagnosis (TRIPOD) guidelines [26].

### Sensitivity analysis

As a sensitivity analysis for external validation, we used data from institutions that were not part of the JIPAD registry between 2018 and 2021 but were newly included in 2022. The logistic regression model developed using the training cohort was applied to this external data set, and the AUC was calculated. To ensure the reliability of the receiver operating characteristic analysis, we excluded facilities with fewer than 10 cases or no tracheostomy events, because AUC estimates in such settings would be unstable or undefined. In another sensitivity analysis, to account for institutional variability, we constructed a generalized linear mixed model (GLMM) with a logistic link, incorporating the institution as a random effect. All fixed-effect covariates were identical to those used in the multivariable logistic regression model previously developed using the training data set. The model’s discrimination performance was evaluated using the AUC.

We also conducted a sensitivity analysis in which the outcome was redefined as tracheostomy during the ICU stay rather than within 14 days of ICU admission. The discriminative performance of the prediction model was assessed using ROC analysis.

### Simplified scoring system development

To enhance the clinical interpretability and bedside applicability of our model, we developed a simplified risk score named the Sepsis Tracheostomy early Prediction (STeP) score, derived from the final multivariable logistic regression model.

After examining the *β* coefficients from the final model, the coefficients were multiplied by four and rounded to the nearest integer to facilitate clinical interpretability and to create an integer-based STeP score. For standardized continuous variables, cutoffs were determined from the original scale using the cohort mean and standard deviation, so that point assignments reflected approximately 1-Standard Deviation (SD) and 2-SD differences from the mean, depending on the direction and magnitude of the *β* coefficient.

In addition, based on the distribution of STeP scores in the training cohort, the patients were stratified into three risk categories using tertile cutoffs, such that the number of patients in each group was approximately equal.

Fine–Gray competing risks regression was performed on the validation cohort (admissions in 2022) to estimate subdistribution hazard ratio (sHR) for tracheostomy, treating both death in the ICU before tracheostomy and discharge alive from the ICU without tracheostomy as competing events [27]. This analysis was conducted using the validation data set without applying exclusion criteria based on ICU death prior to tracheostomy and included only patients with complete data necessary for calculating the STeP score. A univariable model was constructed with the STeP score as a continuous covariate. The sHR and its 95% confidence interval were reported.

### Statistical analysis

Continuous variables are reported as median (interquartile range: IQR) and categorical variables as number (percent). Comparisons of continuous variables between two groups were performed using the Mann–Whitney *U* test, while comparisons of categorical variables were conducted using the chi-square test. To provide context for the discriminative performance of the STeP score, we compared it both with the STeP full model and with conventional ICU severity scores (SOFA, APACHE II) widely used in clinical practice. The AUCs for the STeP model, STeP score, SOFA, and APACHE II in predicting tracheostomy were compared using DeLong’s test. All analyses were carried out using R version 4.4.2 (R Foundation for Statistical Computing, Vienna, Austria), with a significance threshold set at *p* < 0.05. In addition, we developed a web-based application using R Shiny to facilitate individualized risk calculation based on both the final multivariable model (STeP model) and the simplified scoring system (STeP score).

## Results

### Patient characteristics

**Fig. 1:**
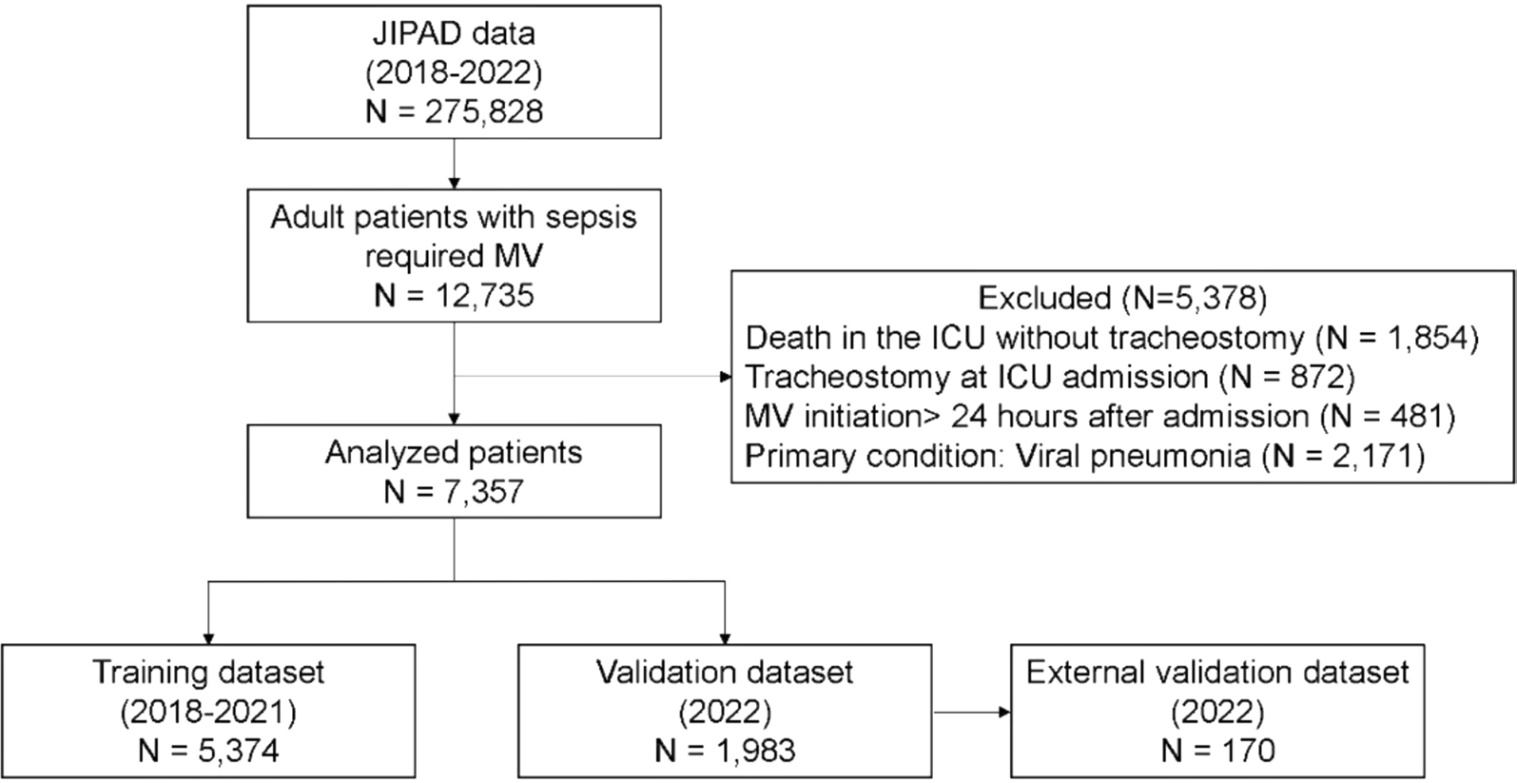
Flowchart of patient selection. *JIPAD* Japanese Intensive care PAtient Database; *MV* mechanical ventilation

The patient flowchart is shown in Fig. 1. Of the 275,828 patients included in the JIPAD database during the study period, 12,735 met the inclusion criteria. Of these, the following were excluded from the analysis: patients who died in the ICU without receiving a tracheostomy (*n* = 1854), those with a tracheostomy at ICU admission (*n* = 872), those who initiated mechanical ventilation more than 24 h after ICU admission (*n* = 481), and those whose primary diagnosis was viral pneumonia (*n* = 2171). Ultimately, 7357 patients were included in the study. Of these, 5374 patients comprised the training cohort and 1983 comprised the validation cohort. The patient characteristics are presented in Table 1.

**Table 1:** Baseline characteristics of the training and validation cohorts

	Training data set *n* = 5374	Validation data set *n* = 1983	*p* value
Age	73.0 (64.0–81.0)	74.0 (64.0–80.0)	0.98
Sex, male	3561 (66.3)	1303 (65.7)	0.66
SOFA score	9.0 (7.0–12.0)	9.0 (7.0–12.0)	0.08
APACHE II score	23.0 (18.0–28.0)	23.0 (18.0–28.0)	0.33
SAPSII score	53.0 (44.0–64.0)	54.0 (44.0–65.0)	0.04
P/F ratio	249 (162–358)	246 (159–361)	0.90
PaCO_2_	39.8 (34.0–46.5)	39.7 (33.8–45.8)	0.24
Lactate	2.2 (1.4–3.7)	2.4 (1.5–4.4)	< 0.01
GCS	14.0 (11.0–15.0)	14.0 (11.0–15.0)	0.70
BMI			
Low (< 18.5)	1167 (21.7)	421 (21.2)	
Normal (18.5–24.9)	2895 (53.9)	1071 (54.0)	
Over (25–29.9)	974 (18.1)	351 (17.7)	
Obesity (> = 30.0)	338 (6.3)	140 (7.1)	
Comorbidity			
Respiratory failure	153 (2.8)	61 (3.1)	0.59
Immunosuppression	680 (12.7)	226 (11.4)	0.15
Metastatic cancer	309 (5.7)	103 (5.2)	0.39
Maintenance dialysis	346 (6.4)	92 (4.6)	< 0.01
Liver cirrhosis	117 (2.2)	34 (1.7)	0.23
ICU readmission	684 (12.7)	229 (11.5)	0.18
Tracheostomy	737 (13.7)	276 (13.9)	0.82

*SOFA* Sequential Organ Failure Assessment; *APACHE II* Acute Physiology and Chronic Health Evaluation II; *SAPS II*, Simplified Acute Physiology Score II; *P/F* partial pressure of arterial oxygen/fraction of inspired oxygen; *PaCO*_*2*_ partial pressure of arterial carbon dioxide; *GCS* Glasgow Coma Scale; *BMI* body mass index; *ICU* intensive care unit

### Development of a prediction model for tracheostomy

**Fig. 2:**
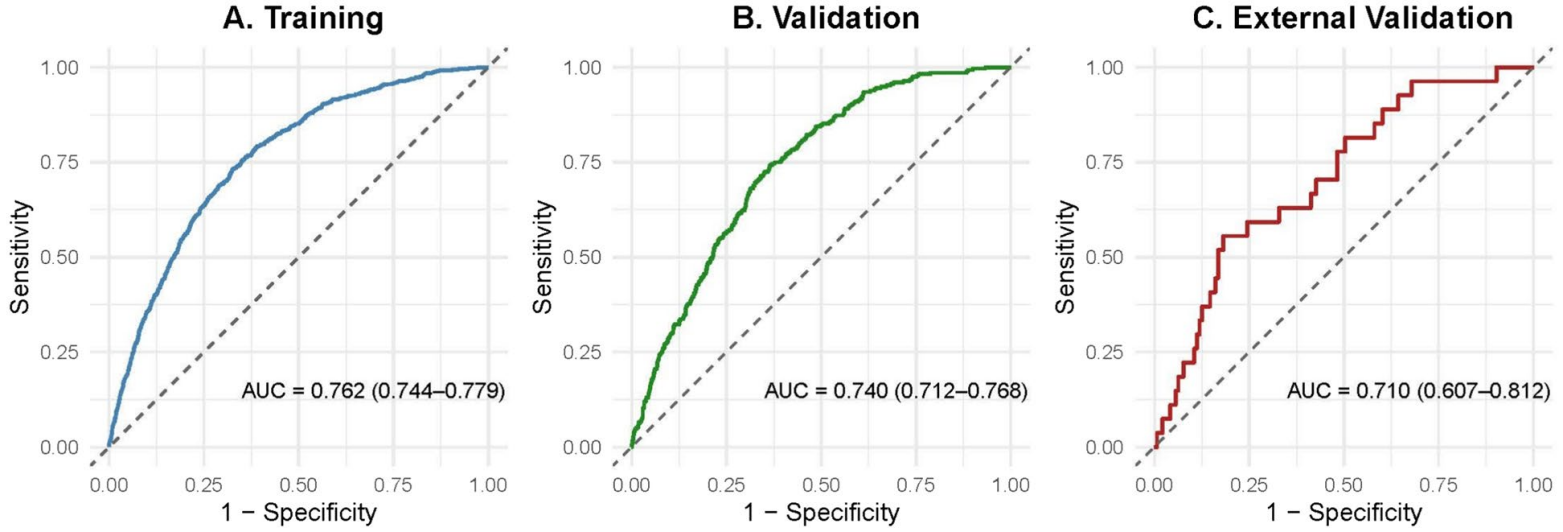
Receiver operating characteristic (ROC) curves of the prediction model in the training and validation cohorts. **A** ROC curve for the training cohort. The blue curve represents the model’s performance, with an AUC of 0.76 (95% CI 0.74–0.78), indicating good discrimination. **B** ROC curve for the temporal validation cohort (2022). The model demonstrated consistent performance, with an AUC of 0.74 (95% CI 0.71–0.77). **C** ROC curve for the external validation cohort, comprising hospitals newly participating in 2022. The model showed acceptable discrimination, with an AUC of 0.71 (95% CI 0.61–0.81), supporting its generalizability across institutions. *ROC* Receiver operating characteristic; *AUC* area under the receiver operating characteristic curve

In total, 5374 patients from 85 facilities were included in the development cohort, of whom 737 (13.7%) underwent tracheostomy within 14 days of ICU admission. To construct the prediction model, we first performed variable selection using LASSO logistic regression with tenfold cross-validation, which identified 8 predictors (Supplementary Figure S1). A multivariable logistic regression model was then fitted using these selected variables. This model was named the STeP model to emphasize its purpose in facilitating early prediction of tracheostomy in ICU patients with sepsis. The STeP model included factors related to patient characteristics (surgical status, BMI of < 18.5 kg/m^2^ and ICU readmission), admission-related variables (admission source and primary diagnosis of respiratory infection), physiological status (Glasgow Coma Scale score), severity scores (APACHE II score), and laboratory data (partial pressure of arterial carbon dioxide [PaCO_2_]). The coefficients of the predictors in the STeP model are illustrated in Supplementary Fig. S2, and the corresponding odds ratios with 95% confidence intervals are summarized in Table 2. The STeP model demonstrated good discriminative performance, with an AUC of 0.76 (95% CI, 0.74–0.78) in the development cohort (Fig. 2).

**Table 2:** Multivariable logistic regression results based on variables selected by LASSO

Variable	Odds Ratio (95% CI)	*p* value
Respiratory infection	2.21 (1.79–2.73)	< 0.01
Admission from in-hospital ward	1.80 (1.48–2.20)	< 0.01
ICU readmission	1.65 (1.33–2.06)	< 0.01
Emergency surgical admission	0.63 (0.47–0.85)	< 0.01
BMI (low)	1.52 (1.26–1.82)	< 0.01
APACHE II	1.26 (1.13–1.39)	< 0.01
GCS	0.87 (0.79–0.95)	< 0.01
PaCO_2_	1.12 (1.05–1.20)	< 0.01

*LASSO* least absolute shrinkage and selection operator; *CI* confidence interval; *ICU* intensive care unit; *GCS* Glasgow Coma Scale; *PaCO*_*2*_ partial pressure of arterial carbon dioxide; *APACHE II* Acute Physiology and Chronic Health Evaluation II; *BMI* body mass index

### Validation of STeP model for tracheostomy

**Fig. 3:**
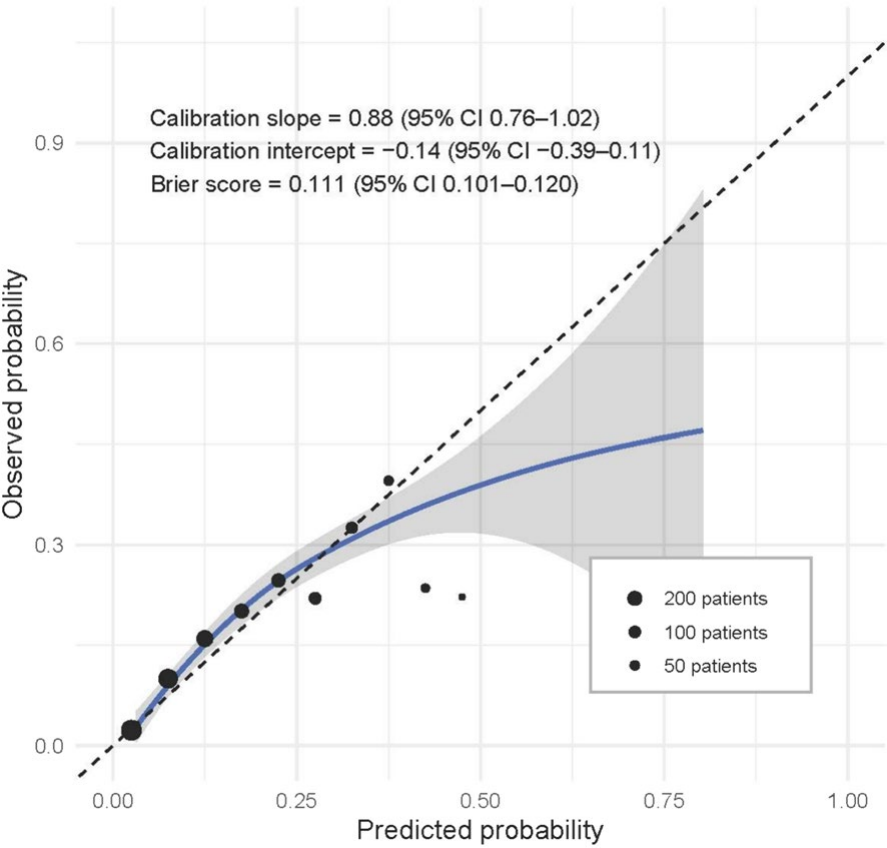
Calibration plot of the STeP model in the validation cohort. The *x*-axis represents the predicted probability of tracheostomy, and the *y*-axis shows the observed proportion of patients who underwent tracheostomy. Black dots indicate the observed event rate within each fixed decile of predicted risk, with dot size reflecting the number of patients in each bin. Dots are shown only for bins containing at least 15 patients. The blue curve represents a locally estimated scatterplot smoothing (LOESS) calibration line, and the shaded gray area denotes the 95% confidence interval. The plot also displays the calibration slope, calibration intercept, and Brier score, each with 95% confidence intervals estimated from 1000 bootstrap samples. *STeP* Sepsis Tracheostomy Early Prediction

The validation cohort consisted of 1983 patients from 89 facilities, of whom 276 (13.9%) underwent tracheostomy. The STeP model was applied to this cohort and yielded an AUC of 0.74 (95% CI, 0.71–0.77), indicating consistent performance (Fig. 2b). Calibration of the STeP model was evaluated using a calibration plot and the Brier score. The calibration plot demonstrated good agreement between predicted and observed outcomes across deciles (Fig. 3). The calibration slope was 0.88 (95% CI, 0.76–1.02), and the intercept was − 0.14 (95% CI, − 0.39–0.11). The Brier score was 0.111 (95% CI, 0.101–0.120), suggesting acceptable overall calibration.

As a sensitivity analysis, we conducted an external validation using data from eight hospitals that began patient registration in the JIPAD registry in 2022. Applying the STeP model, we evaluated its predictive performance in this external validation cohort (8 facilities, *n* = 170) using receiver operating characteristic analysis. The model yielded an AUC of 0.71 (95% CI, 0.61–0.81), indicating good discrimination (Fig. 2c).

In a separate sensitivity analysis, we constructed a GLMM, incorporating hospital-level random intercepts while retaining the same fixed effects as the original model. The GLMM yielded an AUC of 0.81 (Supplementary Figure S3). The standard deviation of the hospital-specific random effects was 0.74, and the intraclass correlation coefficient was 0.14, suggesting a moderate degree of between-hospital variation in tracheostomy rates (Supplementary Table S3).

### Efficacy of the prediction model for tracheostomy in patients with sepsis

Decision curve analysis was performed in the validation cohort, and the resulting plot is shown in Supplementary Figure S4. The analysis demonstrated that the STeP model provided the greatest net benefit across threshold probabilities ranging from 10 to 35%.

### Development of a simplified risk scoring system

**Fig. 4:**
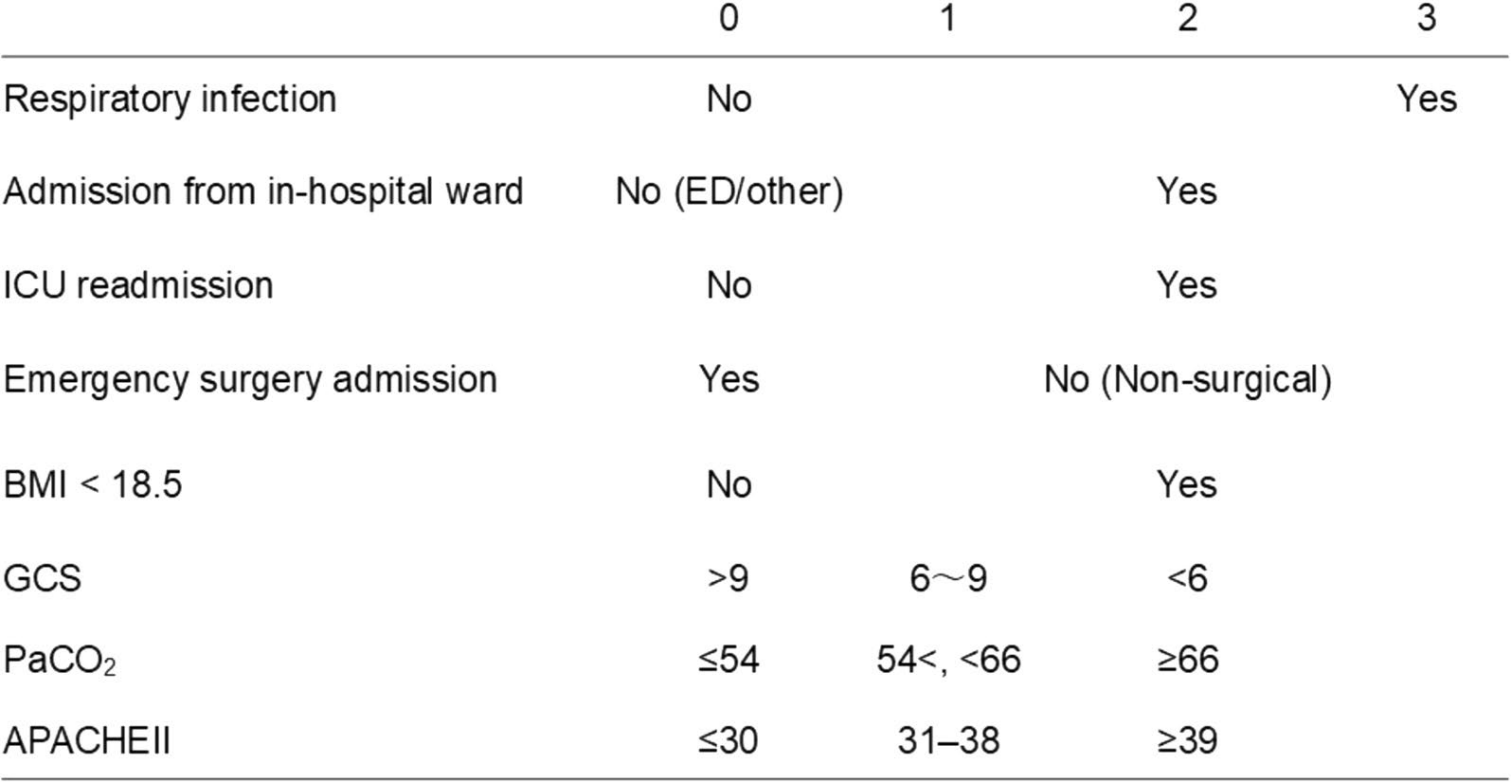
STeP score composition and point allocation. Each of the eight variables included in the simplified STeP score was assigned 0, 1, 2, or 3 points based on the magnitude of the β coefficients from the multivariable logistic regression model and clinical interpretability. The total score ranges from 0 to 17, with higher scores indicating a greater risk of requiring tracheostomy. *STeP* Sepsis Tracheostomy Early Prediction; *ED* emergency department; *ICU* intensive care unit; GCS, Glasgow Coma Scale; *BMI* body mass index; *PaCO*_*2*_ partial pressure of arterial carbon dioxide, *APACHE II* Acute Physiology and Chronic Health Evaluation II

The eight predictors retained in the LASSO-selected full model (STeP model) were ultimately incorporated into the simplified STeP score (Fig. 4).

The resulting STeP score ranged from 0 to 17.

**Fig. 5:**
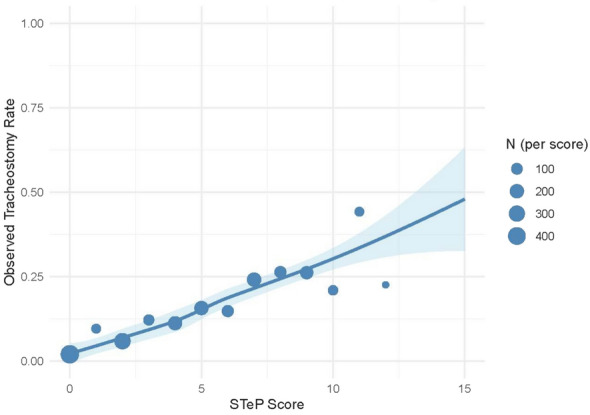
Association between simplified STeP score and tracheostomy rate. This figure illustrates the relationship between the simplified STeP score and the observed proportion of patients who underwent tracheostomy in the validation cohort (2022; *n* = 1983). Each dot represents the tracheostomy rate for a given score, with dot size proportional to the number of patients (only scores with *n* ≥ 10 are shown). The blue line represents a locally estimated scatterplot smoothing (LOESS) curve, with the shaded area indicating the 95% confidence interval. While some individual scores (e.g., score of 10) showed slight deviations, the overall trend was increasing, indicating that the risk of tracheostomy generally rises with higher STeP scores. *STeP* Sepsis Tracheostomy Early Prediction

In the validation cohort, no patients had a score above 15, with the observed range spanning from 0 to 15. The distribution of patients and corresponding tracheostomy rates by score are summarized in Fig. 5. Tracheostomy rates increased in a stepwise manner with higher scores, demonstrating good monotonicity and clinical interpretability. Based on the distribution of STeP scores in the training cohort, patients were stratified into three risk categories: low risk (STeP score of ≤ 2), moderate risk (3–6), and high risk (≥ 7). These cutoffs were chosen to approximate tertile division while maintaining the integer nature of the score. In the validation cohort, the corresponding tracheostomy rates were 4.0% for the low-risk group, 13.6% for the moderate-risk group, and 27.1% for the high-risk group. This scoring system offers a simple and practical tool to estimate tracheostomy risk using routinely available bedside variables. Importantly, the proportion of patients undergoing tracheostomy increased consistently with higher STeP scores, as illustrated in Fig. 5, indicating the model's potential utility in stratifying risk even within the high-risk group. The STeP score demonstrated good discriminative ability in the validation cohort, with an AUC of 0.73 (95% CI, 0.71–0.76) (Supplementary Figure S5), supporting its potential utility as a bedside tool for tracheostomy risk assessment in patients with sepsis based on data available within the first 24 h of ICU admission.

We compared the predictive performance of the STeP score with that of the STeP model (full model) in the validation cohort. Although the difference in AUC between the STeP score (0.73) and the STeP model (AUC = 0.74) was small, it was statistically significant by DeLong’s test (*p* = 0.034). Despite this, the STeP score outperformed conventional severity scores, including SOFA (AUC = 0.56) and APACHE II (AUC = 0.61), for predicting tracheostomy, with these differences also statistically significant by DeLong’s test (*p* < 0.001) (Supplementary Figure S5).

In an additional sensitivity analysis redefining the outcome as tracheostomy during the entire ICU stay, baseline characteristics of patients stratified by tracheostomy status are summarized in Supplementary Table S4. Both the full STeP model and the simplified STeP score demonstrated good discrimination in this analysis, with AUCs of 0.73 and 0.72, respectively (Supplementary Figure S6).

As a sensitivity analysis accounting for the competing risk of death, Fine–Gray regression was performed, treating tracheostomy within 14 days of ICU admission as the event of interest and pre-tracheostomy death and ICU discharge without tracheostomy as competing risks. Patients who remained alive in the ICU without tracheostomy beyond day 14 were censored at that time. The final analysis included *n* = 2337 patients, among whom 276 underwent tracheostomy, 310 died in the ICU before receiving the procedure, and 1496 were discharged alive without tracheostomy. The STeP score remained significantly associated with tracheostomy (sHR, 1.20; 95% CI, 1.17–1.24; *p* < 0.001), even after accounting for the competing risk of death and ICU discharge (Supplementary Table S5). Supplementary Figure S7 illustrates the predicted cumulative incidence of tracheostomy by STeP score, indicating that patients with higher scores had markedly increased cumulative incidence over time.

## Discussion

In patients with sepsis requiring invasive ventilator management in the ICU, we developed a model to predict tracheostomy within 14 days of ICU admission, achieving an AUC of 0.76—generally considered indicative of good performance. The model demonstrated comparable predictive ability in both the temporal validation cohort (AUC = 0.74) and the external validation cohort (AUC = 0.71). This study specifically focuses on patients with sepsis and leverages large-scale, multicenter data to construct an integrated prediction model. In contrast to the earlier study by Rodrigues et al. [16], which identified potential risk factors for tracheostomy in a single-center retrospective cohort using traditional statistical and machine learning approaches, this study utilized a nationwide multicenter data set encompassing more than 7000 patients. The variables selected in the full model included indicators related to respiratory status (PaCO_2_), level of consciousness (Glasgow Coma Scale score), severity score (APACHE II) and whether the respiratory system was the primary site of infection. The lower risk observed in patients admitted for emergency surgery may reflect appropriate source control, such as timely drainage, and the fact that these patients were physiologically stable enough to tolerate surgical intervention. These features are characteristic of sepsis and contribute to the interpretability of the model. The decision curve analysis suggests that the STeP model is most useful when clinicians are considering initiating tracheostomy-related discussions or preparations for patients whose predicted risks are approximately 10–35%. Importantly, these statistical utility ranges should not be interpreted as direct procedural decision thresholds, as clinical practice typically requires higher probability thresholds given tracheostomy’s invasiveness and associated risks. The model is intended primarily as a decision support tool for early family discussions, resource planning, and multidisciplinary care coordination rather than direct procedural guidance.

We not only developed the STeP model—a robust and externally validated prediction model—but also created a simplified risk score (STeP score) and implemented a web-based calculator, providing a practical and generalizable tool for real-time clinical decision-making in sepsis care. While the STeP score demonstrated a slightly lower AUC compared to the full model, its simplicity and ease of use make it a practical tool for bedside application. We established three risk categories for tracheostomy based on the STeP score: low risk (STeP score of ≤ 2), moderate risk (3–6), and high risk (≥ 7). While the high-risk group had a tracheostomy rate of 27.1%, this corresponds to nearly double the overall rate (13.9%) in the validation cohort. Given that tracheostomy is performed selectively and often delayed, an event rate of approximately 30% still represents meaningful risk enrichment and may assist clinicians in prioritizing discussions and preparation for tracheostomy in appropriate patients. The nearly linear relationship between the simplified STeP score and tracheostomy rate (Fig. 5) is highly informative for clinicians. Nevertheless, categorization into three risk groups also has practical advantages, as it simplifies communication, facilitates bedside decision-making, and allows stratified comparisons in research.

Furthermore, the robustness of our findings was supported by a sensitivity analysis redefining the outcome as tracheostomy during the entire ICU stay, which yielded consistent discrimination for both the full model and the simplified STeP score.

Predicting the need for tracheostomy is inherently challenging; however, our findings demonstrate that it can be estimated with reasonable accuracy using indicators available within the first 24 h of ICU admission. This has important implications for clinical practice, including patient communication, ICU bed management, and surgical planning.

Previous studies comparing early and late tracheostomy have yielded inconsistent results, particularly with respect to mortality [28, 29]. This inconsistency may be partly attributable to the lack of appropriate identification of patients who truly require tracheostomy [30]. Specifically, the absence of a robust risk stratification system may have led to the inclusion of heterogeneous patient populations, thereby diluting the potential benefits of early tracheostomy in those most likely to benefit. By enabling early identification of patients most likely to require tracheostomy, our prediction model may help refine patient selection in future studies and guide clinical decisions, potentially clarifying the benefits of timely tracheostomy.

There are several limitations to this study. First, the determination of tracheostomy involves factors, such as anatomical airway narrowing and sputum clearance ability [31], but these data are not included in our current prediction system. While such information would likely improve the accuracy of the tracheostomy prediction model, it is not available in the JIPAD database and is inherently difficult to obtain within the first 24 h of ICU admission. Second, the JIPAD database only captures whether a tracheostomy was performed during the ICU stay, lacking data on procedures conducted after ICU discharge. Third, tracheostomy practices may vary between institutions; the decision to perform the procedure is ultimately at the discretion of the attending physician, and institutional policies differ widely. As such, the model’s performance may vary across settings, and its generalizability should be interpreted in light of this variability. Although the intraclass correlation coefficient of 0.14 indicates moderate inter-facility variation in tracheostomy decisions, our multivariable logistic regression model—constructed without including facility-level information—still demonstrated acceptable predictive performance (AUC = 0.74). Given its simplicity and ease of implementation, especially in contexts, where facility identifiers are unavailable or in newly participating institutions, the STeP model remains a practical tool for early clinical decision-making. Fourth, this study was based on a nationwide Japanese ICU database, which may limit the generalizability of findings to other countries with different healthcare systems, cultural norms, and clinical practices—particularly with respect to the timing and threshold for tracheostomy. Because the decision to perform tracheostomy may be influenced by system-specific factors, such as institutional policies, clinician discretion, and resource availability, the STeP model may reflect practice patterns unique to the Japanese context. As such, caution is warranted when applying the score to international populations. However, the large sample size, inclusion of diverse hospital types, and methodological rigor support the model’s internal validity. Future studies should examine the applicability of the STeP score in international settings to establish its broader applicability. Fifth, the primary outcome in this study—tracheostomy performed within 14 days of ICU admission—may have been influenced by institutional practices, physician discretion, and resource availability, rather than being determined solely by clinical necessity. As a result, our model may reflect prevailing practice patterns rather than true clinical need, potentially limiting its generalizability across diverse clinical environments. Future research should explore alternative outcome definitions that more directly capture the clinical indications for tracheostomy. Sixth, we did not employ machine learning-based modeling approaches in this study. Our primary goal was to create a transparent and interpretable tool that can be applied at the bedside without specialized software. While machine learning methods may provide slightly higher accuracy, they often lack transparency and can be difficult to implement in real time, so we prioritized practicality and clinician trust. Finally, to construct a simple and interpretable logistic regression model using early ICU data, we excluded patients who died in the ICU before undergoing tracheostomy, which may introduce selection bias, because some of these patients might have required tracheostomy had they survived longer. Consequently, the STeP score estimates tracheostomy risk only among patients who lived long enough to be considered for the procedure, potentially limiting its generalizability to all ventilated patients with sepsis. To explore the impact of early death as a competing risk, we conducted a Fine–Gray competing risks analysis using a validation data set that included patients who died before tracheostomy. We employed competing risk analysis rather than traditional survival analysis, because ICU death before tracheostomy and ICU discharge without tracheostomy represent competing events that preclude the procedure during the ICU stay, not merely censoring events. The Fine–Gray model provides clinically interpretable cumulative incidence estimates that account for the reality that critically ill patients face multiple competing outcomes during their ICU stay. This analysis confirmed that the STeP score remained significantly associated with tracheostomy, even after accounting for death as a competing event. These findings support the robustness of the score. Although our subsequent competing risk analysis in the validation cohort addressed this issue to some extent, incorporating such competing risks from the outset may further strengthen methodological robustness. Future studies could consider integrating competing risk frameworks at the model development stage to potentially enhance accuracy and generalizability. Ultimately, it is important to emphasize that the STeP model estimates tracheostomy risk conditional on ICU survival, rather than universally predicting tracheostomy for all ventilated patients. In clinical practice, this means the model is best applied to patients who have survived their initial critical phase, and can inform decisions, such as resource planning and timing of tracheostomy discussions.

Despite these limitations, the practical implementation of our model provides a useful framework for individualized tracheostomy decision-making and lays the groundwork for future interventional studies. Importantly, the STeP score is designed to assist, not replace, individualized clinical decision-making. Clinicians should exercise caution to avoid the inappropriate use of the score—for example, by uniformly recommending early tracheostomy to all high-risk patients or deferring necessary procedures in low-risk individuals. Decisions should always be made in the context of each patient's overall clinical status, prognosis, and preferences, ideally through shared decision-making with patients or their families. To facilitate real-time clinical application, we implemented the developed model as a web-based calculator using the R Shiny platform (Supplementary Figure S8). This tool enables bedside prediction of tracheostomy risk in ICU patients with sepsis and is freely accessible online at https://sepsis-tracheostomy.shinyapps.io/stepscore/. In future research, the prediction model may be used to identify high-risk patients likely to benefit from tracheostomy. Risk stratification using this model could support prospective or interventional studies aimed at evaluating the clinical impact of tracheostomy timing (e.g., early vs. late) in high-risk patient subgroups, ultimately clarifying whether timely tracheostomy may improve outcomes in selected populations.

## Conclusion

In patients with sepsis requiring ventilator management in the ICU, we developed a model for early prediction of the need for tracheostomy, along with a simplified scoring system for practical bedside use. These tools may support timely risk stratification, clinical communication, and resource planning.

## Supplementary Information

Additional file 1 (Supplementary Table 1. Summary of all candidate predictors included in the LASSO regression model)

Additional file 2 (Supplementary Table 2. Proportion of missing data for each variable in the training and validation cohorts)

Additional file 3 (Supplementary Table 3. Summary of hospital-level variation estimated by the GLMM used for sensitivity analysis)

Additional file 4 (Supplementary Table 4. Baseline characteristics of patients stratified by tracheostomy during the entire ICU stay)

Additional file 5 (Supplementary Table 5. Fine–Gray Competing Risks Analysis for ICU Tracheostomy Using the STeP Score)

Additional file 6 (Supplementary Figure 1. Variable importance in the LASSO model and the relationship between λ and the number of selected variables. (A) Cross-validation plot for LASSO logistic regression. The mean binomial deviance is plotted against the logarithm of the regularization parameter (log(λ)). Two vertical dashed lines indicate the λ value that minimizes the mean deviance (lambda.min, left) and the largest λ within one standard error of the minimum (lambda.1se, right). The final model was selected using the one-standard-error rule. Ten-fold cross-validation was used to determine the optimal λ. (B) LASSO regularization path. Each curve represents the coefficient trajectory of one predictor as a function of log(λ). Predictors included in the final model based on the one-standard-error rule are highlighted in red. (C) Coefficients of predictors selected by the LASSO model. Bars represent the magnitude and direction of each variable’s contribution to the model. Positive coefficients are shown in blue; negative coefficients are shown in red. LASSO, least absolute shrinkage and selection operator; APACHE II, Acute Physiology and Chronic Health Evaluation II; ICU, intensive care unit; BMI, body mass index; PaCO2, partial pressure of arterial carbon dioxide; GCS, Glasgow Coma Scale)

Additional file 7 (Supplementary Figure 2. Coefficients of variables selected in the final multivariable logistic regression model. (A) Coefficients of Selected Variables. This bar plot displays the coefficients of 8 predictors selected in the final multivariable logistic regression model (STeP model). Variables with positive coefficients, indicating an increased likelihood of tracheostomy, are shown in red. Variables with negative coefficients, associated with a decreased likelihood of tracheostomy, are shown in blue. Coefficient values represent the magnitude and direction of each variable’s contribution to the prediction model. (B) Mean and Standard Deviation of Continuous Variables. This table displays the mean and standard deviation for the continuous variables included in the final multivariable logistic regression model (STeP model). These continuous variables were standardized using z-score normalization (subtracting the mean and dividing by the standard deviation) before fitting the model. ICU, intensive care unit; BMI, body mass index; APACHE II, Acute Physiology and Chronic Health Evaluation II; PaCO2, partial pressure of arterial carbon dioxide; GCS, Glasgow Coma Scale, STeP, Sepsis Tracheostomy Early Prediction)

Additional file 8 (Supplementary Figure 3. ROC curve of the GLMM accounting for between-hospital variability. This ROC curve illustrates the performance of the GLMM, which incorporated hospital-level random intercepts to account for between-hospital variability. The model demonstrated good discriminative ability for predicting tracheostomy, with an AUC of 0.81 (95% CI, 0.79–0.84). The curve was generated using the validation data set that included hospital identifiers. ROC, Receiver operating characteristic; GLMM, generalized linear mixed model; AUC, area under the receiver operating characteristic curve)

Additional file 9 (Supplementary Figure 4. Decision curve analysis of the tracheostomy prediction model in the validation cohort. This decision curve analysis illustrates the net benefit of the tracheostomy prediction model across a range of threshold probabilities in the validation cohort. The red curve represents the net benefit of the model, and the shaded area indicates its 95% confidence interval (CI), estimated using 1000 bootstrap resamples. The model demonstrates the greatest net benefit within the threshold range of 10–35%, suggesting it is most effective for informing tracheostomy-related clinical decisions within this probability range.)

Additional file 10 (Supplementary Figure 5. Comparison of ROC curves for the STeP score, STeP full model, SOFA, and APACHE II in the validation cohort. These ROC curves illustrate the discriminative performance of the simplified STeP score, the STeP model (full model), SOFA, and APACHE II for predicting tracheostomy in the validation cohort. The AUC of the STeP score was 0.73 (95% CI, 0.71–0.76), indicating good discrimination. While the AUC of the STeP score was slightly lower than that of the STeP model (AUC: 0.740, 95% CI, 0.71–0.77), the difference was statistically significant by DeLong’s test (p = 0.034). The STeP score outperformed both SOFA (AUC: 0.56, 95% CI, 0.52–0.59) and APACHE II (AUC: 0.61, 95% CI, 0.58–0.65) (p < 0.05 for each comparison by DeLong’s test). ROC, receiver operating characteristic; STeP, Sepsis Tracheostomy Early Prediction; AUC, area under the receiver operating characteristic curve; SOFA, Sequential Organ Failure Assessment; APACHE, Acute Physiology and Chronic Health Evaluation.)

Additional file 11 (Supplementary Figure 6. ROC curves for the sensitivity analysis using an alternative outcome definition (tracheostomy during entire ICU stay). Receiver operating characteristic (ROC) curves of the full STeP model and the simplified STeP score are shown. The AUCs were 0.73 for the full model and 0.72 for the simplified score, both indicating good discrimination.)

Additional file 12 (Supplementary Figure 7. Cumulative incidence of tracheostomy according to STeP score, estimated using Fine–Gray regression. The figure shows the predicted cumulative incidence function (CIF) of tracheostomy during ICU stay, stratified by STeP score groups (0, 3, 6, 9, 12, and 15). The analysis was conducted using a Fine–Gray competing risks model, treating both ICU death and ICU discharge without tracheostomy as competing events. A higher STeP score was associated with a higher cumulative incidence of tracheostomy.)

Additional file 13 (Supplementary Figure 8. Screenshot of the web-based STeP calculator (full model and score). (A) Web-based calculator for the STeP model (full model). The full model (STeP model), which calculates the predicted probability of tracheostomy based on 8 variables selected through LASSO logistic regression. (B) Web-based calculator for the STeP Score. The simplified scoring system (STeP score), which includes eight variables and yields a total score ranging from 0 to 17. Based on the score distribution in the training cohort, patients are stratified into three risk categories: low risk (STeP score of ≤2), moderate risk (3–6), and high risk (≥7). ICU, intensive care unit; STeP, Sepsis Tracheostomy Early Prediction; BMI, body mass index; GCS, Glasgow Coma Scale; PaCO2, partial pressure of arterial carbon dioxide; APACHE II, Acute Physiology and Chronic Health Evaluation II.)

## Data Availability

The data sets used in this study are not publicly available because of restrictions imposed by the JIPAD working group. However, the application developed for risk score calculation is publicly accessible at https://sepsis-tracheostomy.shinyapps.io/stepscore/. This application is based on the predictive model developed in the study and is currently intended for research and educational purposes. While it may serve as a reference to support clinical decision-making, it should not be used as the sole basis for independent clinical decisions without further validation.

## Abbreviations

APACHE II: Acute Physiology and Chronic Health Evaluation II
AUC: Area under the curve
BMI: Body mass index
CI: Confidence interval
GLMM: Generalized linear mixed model
HCU: High care unit
ICU: Intensive care unit
JIPAD: Japanese Intensive care PAtient Database
LASSO: Least absolute shrinkage and selection operator
P/F: Partial pressure of arterial oxygen/fraction of inspired oxygen
PaCO_2_: Partial pressure of arterial carbon dioxide
ROC: Receiver operating characteristic
SAPS II: Simplified Acute Physiology Score II
sHR: Subdistribution hazard ratio
SOFA: Sequential Organ Failure Assessment
STeP: Sepsis Tracheostomy Early Prediction
TRIPOD: Transparent Reporting of a Multivariable Prediction Model for Individual Prognosis or Diagnosis
