# Supplementary material for: Development and validation of the STeP score for predicting tracheostomy in patients with sepsis using a nationwide ICU database: a retrospective observational study
Source: J Intensive Care. 2025 Nov 14;13:64. doi: 10.1186/s40560-025-00833-8 (PMC12619163; doi:10.1186/s40560-025-00833-8)

Supplementary Figure 1

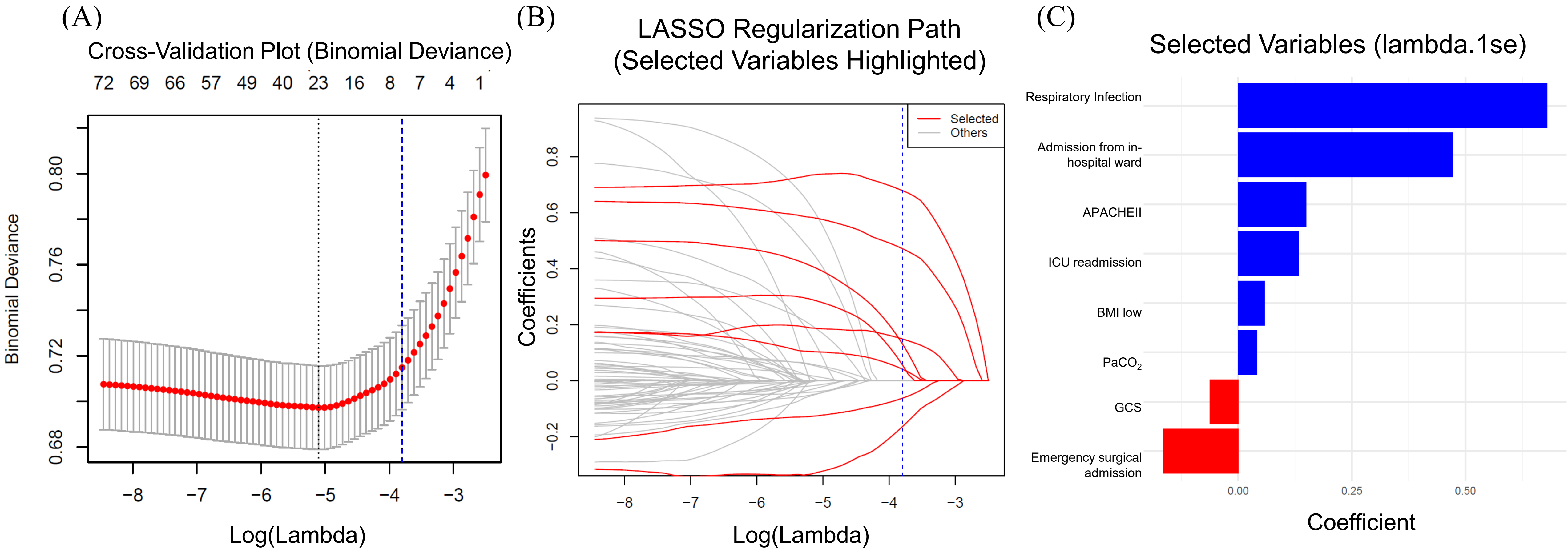

Supplement: Supplementary file 6 — Additional file 6 (Supplementary Figure 1. Variable importance in the LASSO model and the relationship between λ and the number of selected variables. (A) Cross-validation plot for LASSO logistic regression. The mean binomial deviance is plotted against the logarithm of the regularization parameter (log(λ)). Two vertical dashed lines indicate the λ value that minimizes the mean deviance (lambda.min, left) and the largest λ within one standard error of the minimum (lambda.1se, right). The final model was selected using the one-standard-error rule. Ten-fold cross-validation was used to determine the optimal λ. (B) LASSO regularization path. Each curve represents the coefficient trajectory of one predictor as a function of log(λ). Predictors included in the final model based on the one-standard-error rule are highlighted in red. (C) Coefficients of predictors selected by the LASSO model. Bars represent the magnitude and direction of each variable’s contribution to the model. Positive coefficients are shown in blue; negative coefficients are shown in red. LASSO, least absolute shrinkage and selection operator; APACHE II, Acute Physiology and Chronic Health Evaluation II; ICU, intensive care unit; BMI, body mass index; PaCO2, partial pressure of arterial carbon dioxide; GCS, Glasgow Coma Scale) [file 40560_2025_833_MOESM6_ESM.pdf]
