# Supplementary material for: Development and validation of the STeP score for predicting tracheostomy in patients with sepsis using a nationwide ICU database: a retrospective observational study
Source: J Intensive Care. 2025 Nov 14;13:64. doi: 10.1186/s40560-025-00833-8 (PMC12619163; doi:10.1186/s40560-025-00833-8)
Supplement: Supplementary file 7 — Additional file 7 (Supplementary Figure 2. Coefficients of variables selected in the final multivariable logistic regression model. (A) Coefficients of Selected Variables. This bar plot displays the coefficients of 8 predictors selected in the final multivariable logistic regression model (STeP model). Variables with positive coefficients, indicating an increased likelihood of tracheostomy, are shown in red. Variables with negative coefficients, associated with a decreased likelihood of tracheostomy, are shown in blue. Coefficient values represent the magnitude and direction of each variable’s contribution to the prediction model. (B) Mean and Standard Deviation of Continuous Variables. This table displays the mean and standard deviation for the continuous variables included in the final multivariable logistic regression model (STeP model). These continuous variables were standardized using z-score normalization (subtracting the mean and dividing by the standard deviation) before fitting the model. ICU, intensive care unit; BMI, body mass index; APACHE II, Acute Physiology and Chronic Health Evaluation II; PaCO2, partial pressure of arterial carbon dioxide; GCS, Glasgow Coma Scale, STeP, Sepsis Tracheostomy Early Prediction) [file 40560_2025_833_MOESM7_ESM.pdf]

Supplementary Figure 2

(A)

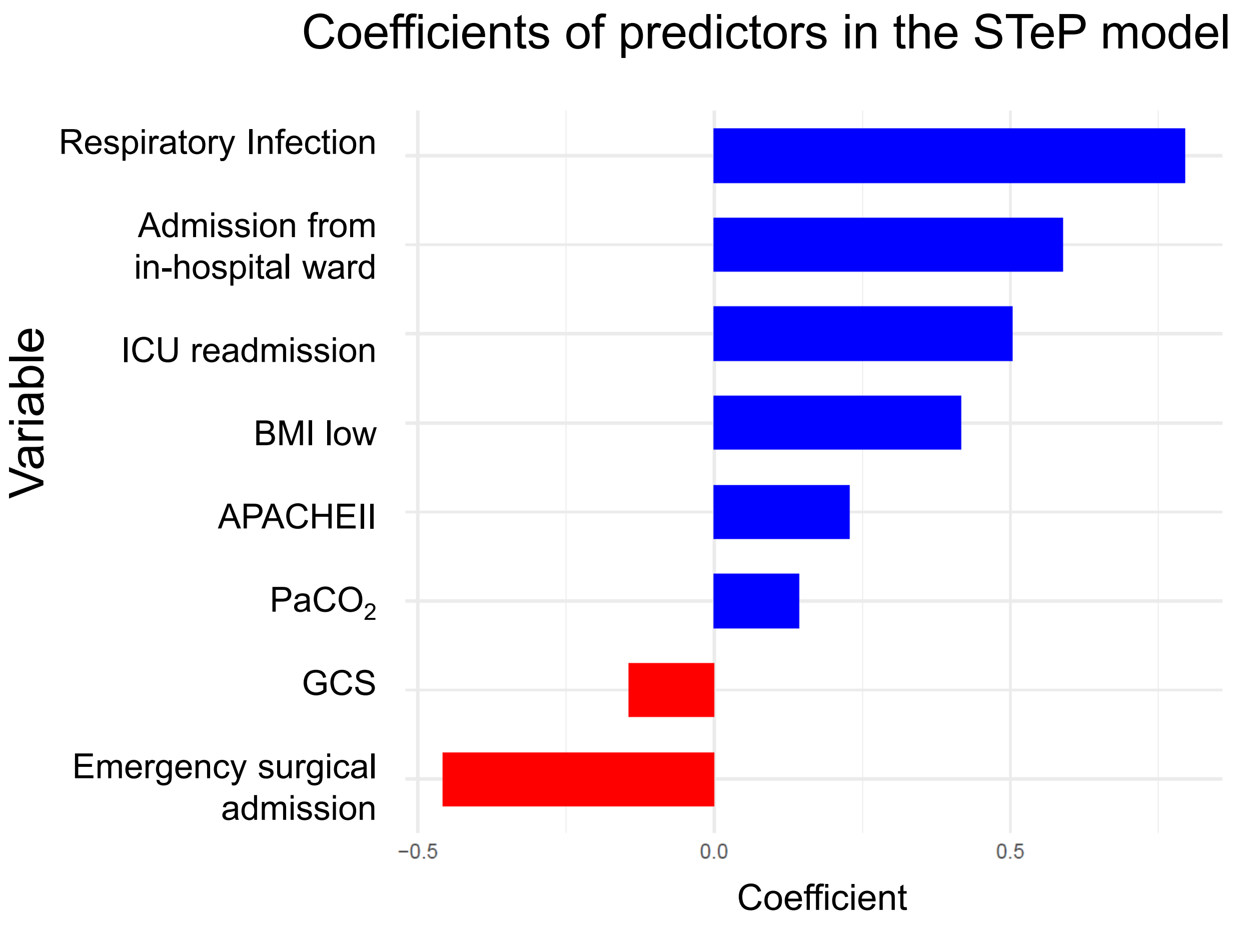

(B)

Mean and Standard Deviation of Continuous Variables

Variable	Mean	Standard Deviation
APACHEII	23.28	7.48
$PaCO_2$	41.54	12.41
GCS	12.41	3.74
