# Supplementary figures and images for: Development and validation of the STeP score for predicting tracheostomy in patients with sepsis using a nationwide ICU database: a retrospective observational study
Source: J Intensive Care. 2025 Nov 14;13:64. doi: 10.1186/s40560-025-00833-8 (PMC12619163; doi:10.1186/s40560-025-00833-8)

**Supplementary Figure 3**

**GLMM ROC Curve (Hospital Random Effects)**

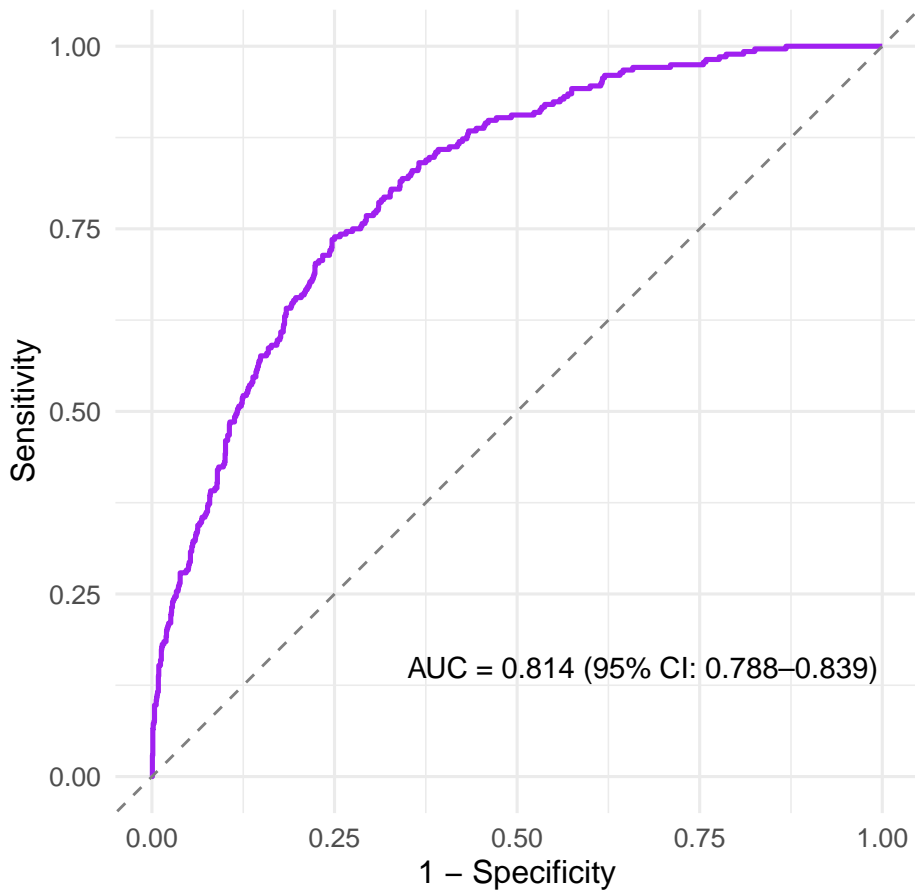

Supplement: Supplementary file 8 — Additional file 8 (Supplementary Figure 3. ROC curve of the GLMM accounting for between-hospital variability. This ROC curve illustrates the performance of the GLMM, which incorporated hospital-level random intercepts to account for between-hospital variability. The model demonstrated good discriminative ability for predicting tracheostomy, with an AUC of 0.81 (95% CI, 0.79–0.84). The curve was generated using the validation data set that included hospital identifiers. ROC, Receiver operating characteristic; GLMM, generalized linear mixed model; AUC, area under the receiver operating characteristic curve) [file 40560_2025_833_MOESM8_ESM.pdf]

Supplementary Figure 5

## ROC Curve Comparison (Validation Dataset)

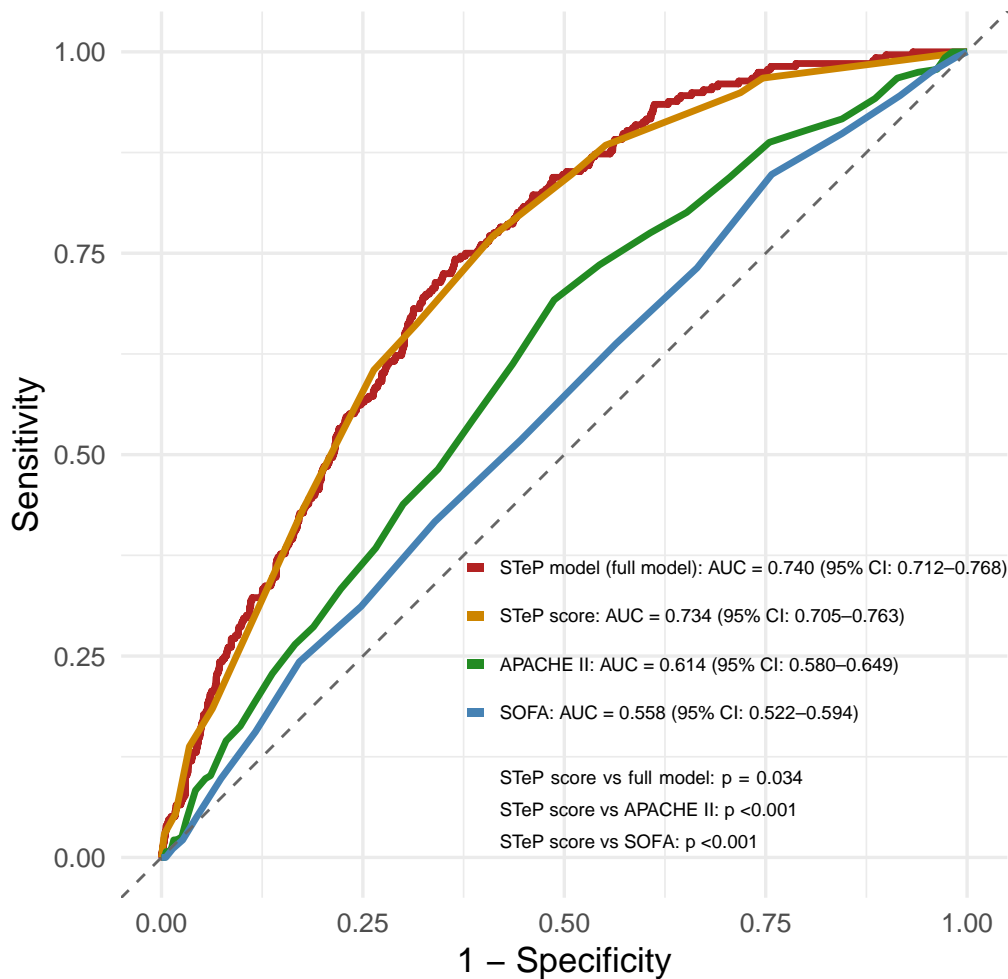

Supplement: Supplementary file 10 — Additional file 10 (Supplementary Figure 5. Comparison of ROC curves for the STeP score, STeP full model, SOFA, and APACHE II in the validation cohort. These ROC curves illustrate the discriminative performance of the simplified STeP score, the STeP model (full model), SOFA, and APACHE II for predicting tracheostomy in the validation cohort. The AUC of the STeP score was 0.73 (95% CI, 0.71–0.76), indicating good discrimination. While the AUC of the STeP score was slightly lower than that of the STeP model (AUC: 0.740, 95% CI, 0.71–0.77), the difference was statistically significant by DeLong’s test (p = 0.034). The STeP score outperformed both SOFA (AUC: 0.56, 95% CI, 0.52–0.59) and APACHE II (AUC: 0.61, 95% CI, 0.58–0.65) (p < 0.05 for each comparison by DeLong’s test). ROC, receiver operating characteristic; STeP, Sepsis Tracheostomy Early Prediction; AUC, area under the receiver operating characteristic curve; SOFA, Sequential Organ Failure Assessment; APACHE, Acute Physiology and Chronic Health Evaluation.) [file 40560_2025_833_MOESM10_ESM.pdf]
