# Supplementary material for: Development and validation of the STeP score for predicting tracheostomy in patients with sepsis using a nationwide ICU database: a retrospective observational study
Source: J Intensive Care. 2025 Nov 14;13:64. doi: 10.1186/s40560-025-00833-8 (PMC12619163; doi:10.1186/s40560-025-00833-8)

Supplementary Figure 4. Decision curve analysis of the tracheostomy prediction model in the validation cohort

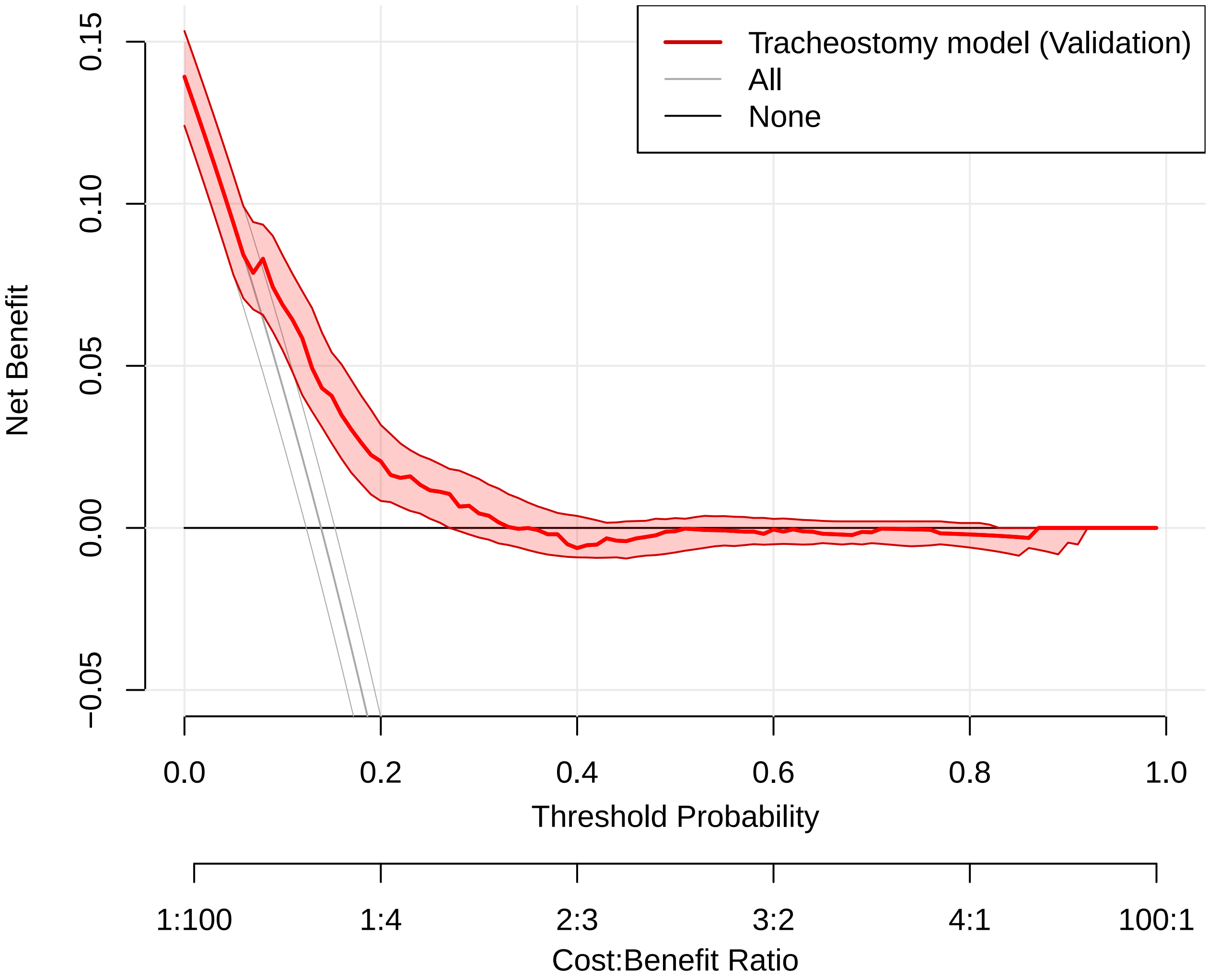

Supplement: Supplementary file 9 — Additional file 9 (Supplementary Figure 4. Decision curve analysis of the tracheostomy prediction model in the validation cohort. This decision curve analysis illustrates the net benefit of the tracheostomy prediction model across a range of threshold probabilities in the validation cohort. The red curve represents the net benefit of the model, and the shaded area indicates its 95% confidence interval (CI), estimated using 1000 bootstrap resamples. The model demonstrates the greatest net benefit within the threshold range of 10–35%, suggesting it is most effective for informing tracheostomy-related clinical decisions within this probability range.) [file 40560_2025_833_MOESM9_ESM.pdf]
