# Supplementary material for: Development and validation of the STeP score for predicting tracheostomy in patients with sepsis using a nationwide ICU database: a retrospective observational study
Source: J Intensive Care. 2025 Nov 14;13:64. doi: 10.1186/s40560-025-00833-8 (PMC12619163; doi:10.1186/s40560-025-00833-8)

Supplementary Figure 6.

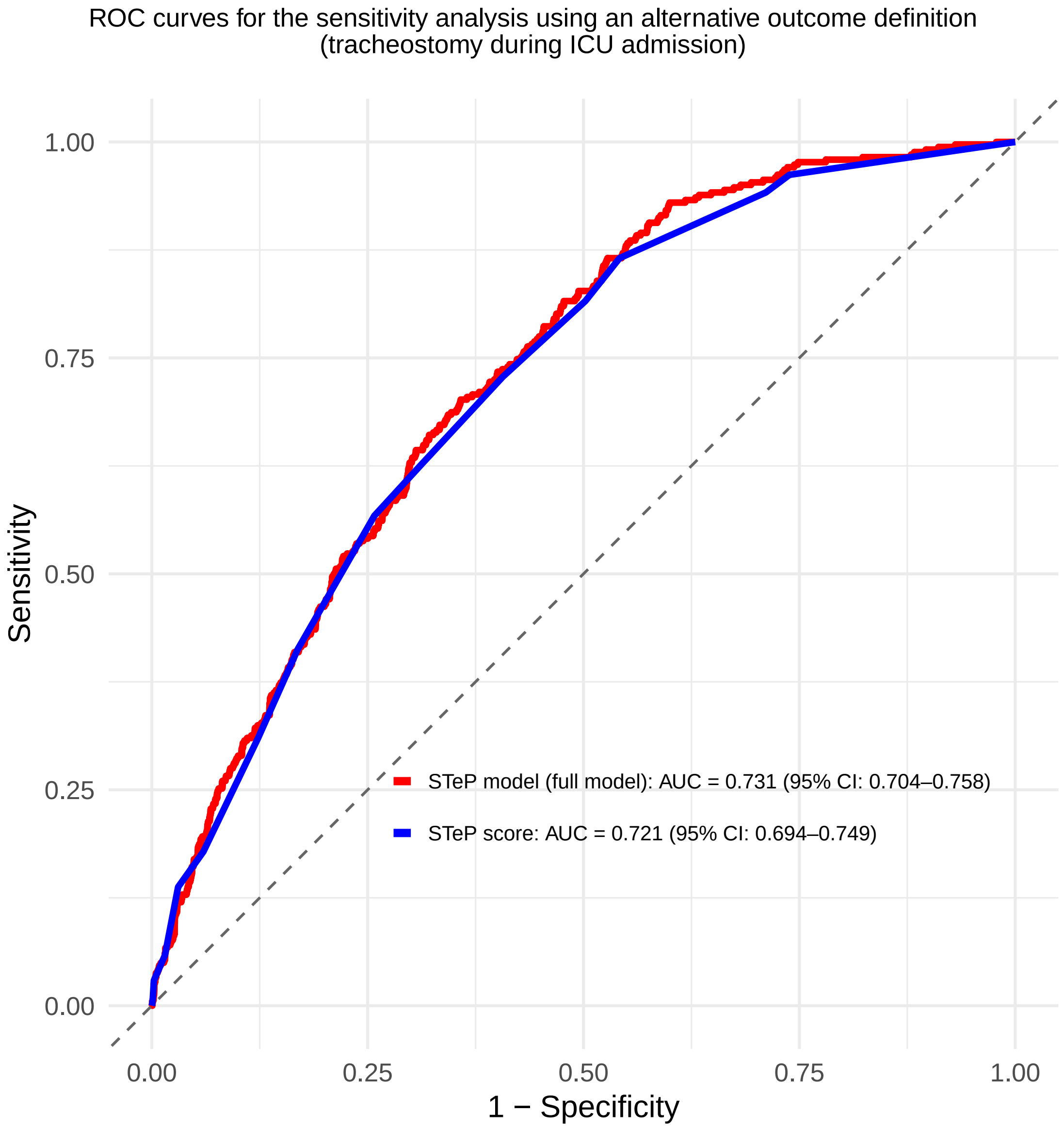

Supplement: Supplementary file 11 — Additional file 11 (Supplementary Figure 6. ROC curves for the sensitivity analysis using an alternative outcome definition (tracheostomy during entire ICU stay). Receiver operating characteristic (ROC) curves of the full STeP model and the simplified STeP score are shown. The AUCs were 0.73 for the full model and 0.72 for the simplified score, both indicating good discrimination.) [file 40560_2025_833_MOESM11_ESM.pdf]
