# Supplementary material for: Development and validation of the STeP score for predicting tracheostomy in patients with sepsis using a nationwide ICU database: a retrospective observational study
Source: J Intensive Care. 2025 Nov 14;13:64. doi: 10.1186/s40560-025-00833-8 (PMC12619163; doi:10.1186/s40560-025-00833-8)

Supplementary Figure 7. Cumulative Incidence of ICU Tracheostomy by STeP Score

Fine–Gray model (n=2337, tracheostomy=276, competing risks=1806, Censored=255)

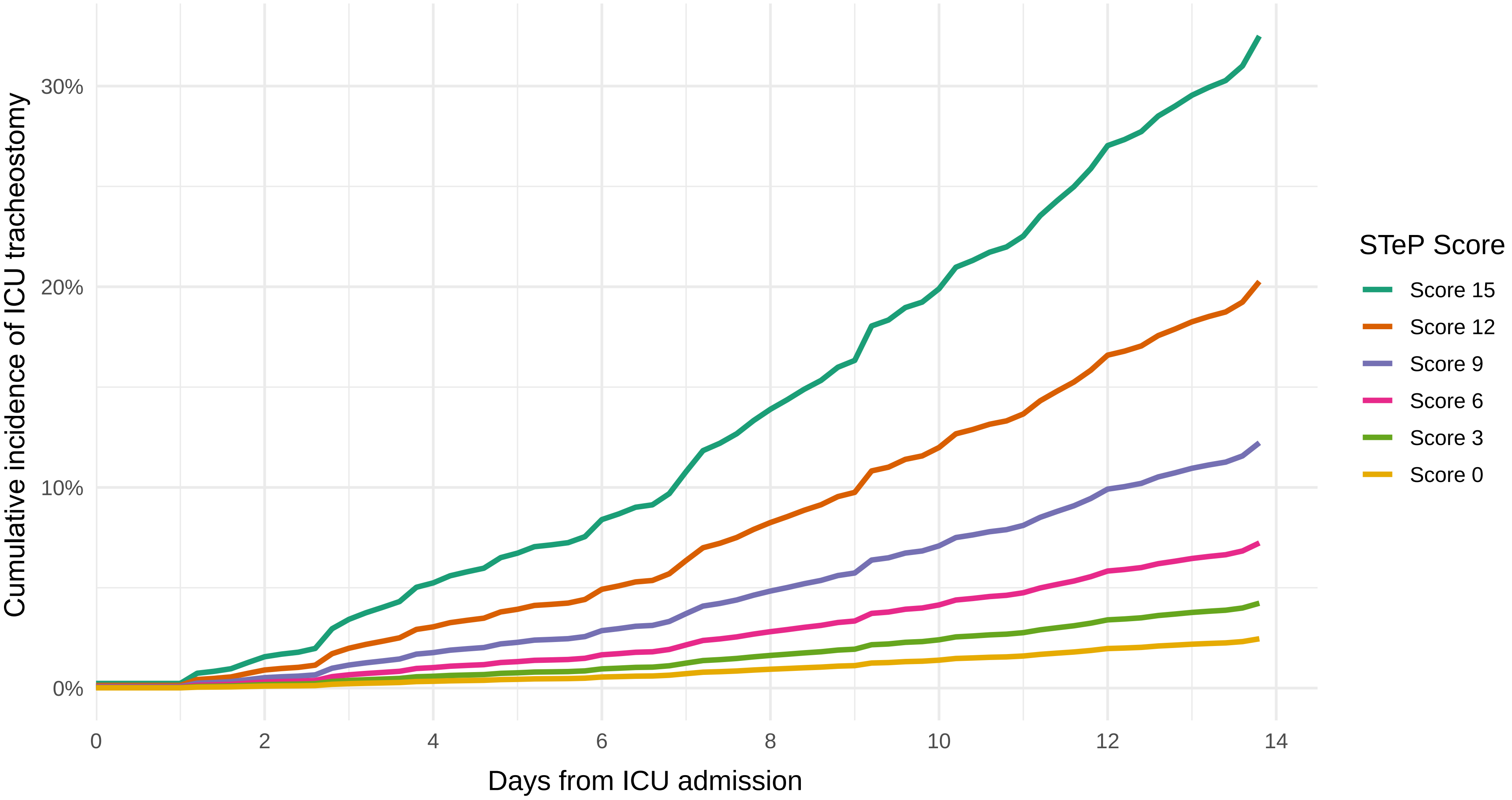

Supplement: Supplementary file 12 — Additional file 12 (Supplementary Figure 7. Cumulative incidence of tracheostomy according to STeP score, estimated using Fine–Gray regression. The figure shows the predicted cumulative incidence function (CIF) of tracheostomy during ICU stay, stratified by STeP score groups (0, 3, 6, 9, 12, and 15). The analysis was conducted using a Fine–Gray competing risks model, treating both ICU death and ICU discharge without tracheostomy as competing events. A higher STeP score was associated with a higher cumulative incidence of tracheostomy.) [file 40560_2025_833_MOESM12_ESM.pdf]
