# Supplementary material for: Development and validation of the STeP score for predicting tracheostomy in patients with sepsis using a nationwide ICU database: a retrospective observational study
Source: J Intensive Care. 2025 Nov 14;13:64. doi: 10.1186/s40560-025-00833-8 (PMC12619163; doi:10.1186/s40560-025-00833-8)

Supplementary Figure 8. Screenshot of the web-based STeP calculator (full model & score)

(A) STeP model (full model)

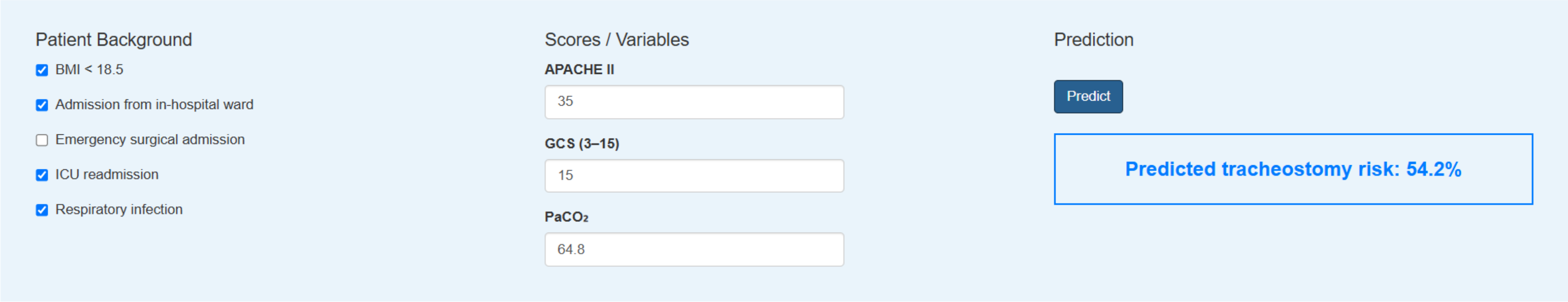

(B) STeP score

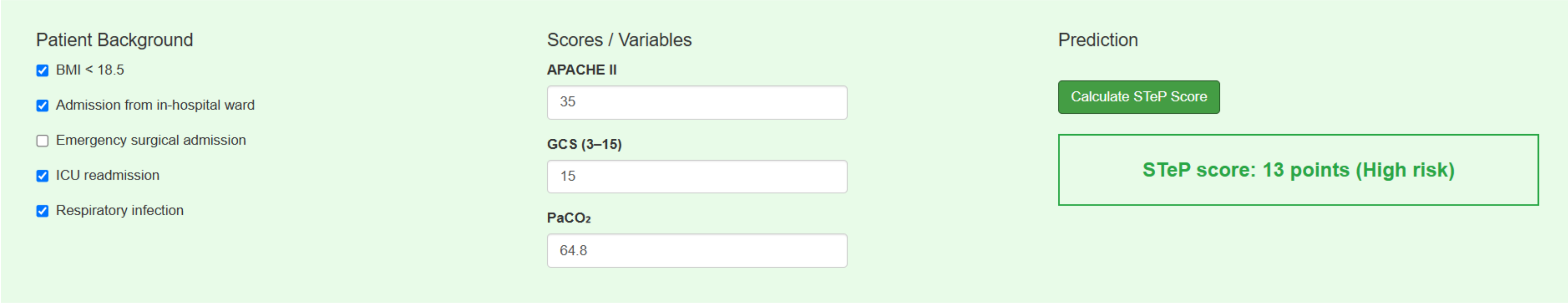

Supplement: Supplementary file 13 — Additional file 13 (Supplementary Figure 8. Screenshot of the web-based STeP calculator (full model and score). (A) Web-based calculator for the STeP model (full model). The full model (STeP model), which calculates the predicted probability of tracheostomy based on 8 variables selected through LASSO logistic regression. (B) Web-based calculator for the STeP Score. The simplified scoring system (STeP score), which includes eight variables and yields a total score ranging from 0 to 17. Based on the score distribution in the training cohort, patients are stratified into three risk categories: low risk (STeP score of ≤2), moderate risk (3–6), and high risk (≥7). ICU, intensive care unit; STeP, Sepsis Tracheostomy Early Prediction; BMI, body mass index; GCS, Glasgow Coma Scale; PaCO2, partial pressure of arterial carbon dioxide; APACHE II, Acute Physiology and Chronic Health Evaluation II.) [file 40560_2025_833_MOESM13_ESM.pdf]
